# Engineering the Melanocortin-4 Receptor to Control Constitutive and Ligand-Mediated G_s_ Signaling *In Vivo*

**DOI:** 10.1371/journal.pone.0000668

**Published:** 2007-08-01

**Authors:** Supriya Srinivasan, Pamela Santiago, Cecile Lubrano, Christian Vaisse, Bruce R. Conklin

**Affiliations:** 1 Gladstone Institute of Cardiovascular Disease, University of California at San Francisco, San Francisco, California, United States of America; 2 Department of Medicine, University of California at San Francisco, San Francisco, California, United States of America; 3 The Diabetes Center, University of California at San Francisco, San Francisco, California, United States of America; Stanford University School of Medicine, United States of America

## Abstract

The molecular and functional diversity of G protein–coupled receptors is essential to many physiological processes. However, this diversity presents a significant challenge to understanding the G protein–mediated signaling events that underlie a specific physiological response. To increase our understanding of these processes, we sought to gain control of the timing and specificity of G_s_ signaling *in vivo*. We used naturally occurring human mutations to develop two G_s_-coupled engineered receptors that respond solely to a synthetic ligand (RASSLs). Our G_s_-coupled RASSLs are based on the melanocortin-4 receptor, a centrally expressed receptor that plays an important role in the regulation of body weight. These RASSLs are not activated by the endogenous hormone α-melanocyte-stimulating hormone but respond potently to a selective synthetic ligand, tetrahydroisoquinoline. The RASSL variants reported here differ in their intrinsic basal activities, allowing the separation of the effects of basal signaling from ligand-mediated activation of the G_s_ pathway *in vivo*. These RASSLs can be used to activate G_s_ signaling in any tissue, but would be particularly useful for analyzing downstream events that mediate body weight regulation in mice. Our study also demonstrates the use of human genetic variation for protein engineering.

## Introduction

G protein–coupled receptors (GPCRs) are the largest known family of cell-surface receptors, encompassing ∼350 distinct members in mammals [1]. GPCRs play key roles in cellular signaling to regulate many important physiological processes, including cellular differentiation [2], immune response [3], smell [4], taste [5], vision [6], heart rate regulation [7], learning and memory [8], and energy homeostasis [9], [10]. They are stimulated by natural ligands, such as light, odorants, biogenic amines, lipids, and peptide hormones. Upon activation, GPCRs transduce signals by coupling to G proteins that modulate the intracellular concentrations of second messenger systems, such as cAMP, Ca^2+^, and phospholipids, to effect sustained changes in transcription and posttranslational modifications and ultimately alter the physiology and behavior of an organism [11].

The diversity of receptors, ligands and G protein effector systems that makes GPCRs biologically important also complicates studies of their function *in vivo*. Standard methods for studying the effects of a GPCR involve the administration of a ligand into a tissue of interest. However, in a whole animal, receptor activation cannot be restricted to a specific cell-type within a particular tissue. In addition, several GPCRs belong to subfamilies that are stimulated by the same ligand, but activate different downstream pathways by coupling to different G protein families [12]. Finally, the difficulty of monitoring the concentrations of endogenous ligands in the tissue of interest has complicated the interpretation of the studies of GPCR activation *in vivo*.

To circumvent these problems, we and others have developed receptors called RASSLs, for Receptors Activated Solely by a Synthetic Ligand; [9], [13], [14]. These engineered receptors are insensitive to their natural endogenous ligands but can still be activated by synthetic agonists. RASSL design is based on the premise that endogenous peptide hormones bind at different sites on the cognate receptors than do small-molecule synthetic ligands. Such engineered receptors can be used to activate a G protein pathway of interest rapidly and reversibly, mimicking the speed, localization, regulation, and amplification of endogenous GPCR signals. The resulting cellular and physiological changes are then clearly attributable to the effects of the G protein pathway activated by the RASSL. The first RASSL (RASSL opioid 1 or Ro1) is G_i_-coupled and based on the kappa opioid receptor [15]. Ro1 is unresponsive to physiological concentrations of opioid peptides but is activated by nanomolar concentrations of the selective synthetic ligand spiradoline. It has been used to study the physiological effects of the G_i_ pathway in heart rate variability [16], taste sensation [17], and olfactory perception [18]. We have now generated G_s_-coupled RASSLs based on the melanocortin-4 receptor (MC4R) for the study of G_s_-mediated pathways *in vivo*.

The melanocortin receptor family consists of five members (MC1R–MC5R) that are expressed in diverse mammalian tissues and are involved in a wide range of physiological processes, including the control of pigmentation, adrenal gland function, inflammation, and energy homeostasis [19]. The melanocortin-4 receptor (MC4R) is primarily expressed in the mammalian brain, plays an important role in body weight regulation [20], and is a lead target for the treatment of obesity [21]. MC4R is stimulated by the endogenous ligand α-melanocyte-stimulating hormone (α-MSH), transduces signal through the G_s_ protein, and activates adenylyl cyclase to increase intracellular cAMP. MC4R also has intrinsic basal activity that is suppressed by the inverse agonist agouti-related peptide (AGRP) [22]. Numerous factors make MC4R an excellent choice for G_s_ RASSL development. First, it is exclusively coupled to G_s_ at physiologically relevant concentrations of agonist [19], thus the effects of receptor activation can be clearly attributed to the G_s_ pathway. Second, studies of naturally occurring loss-of-function mutations in MC4R from obese subjects [23]–[27], in conjunction with structure-function studies [28], [29], have identified several key regions of the receptor that are required for binding of the endogenous ligand, α-MSH. Next, the role of MC4R in the regulation of body weight has generated the interest of pharmaceutical companies that have created potent small-molecule agonists of MC4R to treat obesity and can be used to activate an MC4R-based RASSL. Finally, AGRP and other synthetic antagonists, such as SHU9119 [30], [31], can be administered to turn off the RASSL signal *in vivo*.

Here we report the generation of two MC4R-based RASSLs and their properties with respect to their constitutive activities, the potencies of endogenous and synthetic ligands, and their internalization properties. These RASSLs will be a powerful tool to study the downstream effects of constitutive and ligand-mediated G_s_-signaling *in vivo*.

## Materials and Methods

### MC4R Cloning and Mutagenesis

Wild-type MC4R was sequenced as described [32] and cloned into the vector pcDNA 3.1 (Invitrogen, San Diego, CA). The prolactin signal peptide, FLAG epitope tag, and green fluorescent protein were added to the N-terminus of wild-type *MC4R* by standard recombinant DNA methods, and all experiments described were conducted using this construct as the wild-type control, since α-MSH and THIQ showed the same efficacy and potency at this receptor as at the MC4R cDNA alone. Point mutations in *MC4R* were introduced with a site-directed mutagenesis kit (QuikChange, Stratagene, La Jolla, CA) and sequenced to confirm the substitutions. The DNA sequences corresponding to wild-type MC4R, Rm1, and Rm2 can be found at http://www.gladstone.ucsf.edu/gladstone/php/?sitename = conklin.

### Cell Culture, Transfection, and the Generation of Stable Cell Lines

HEK293 cells were maintained in Dulbecco's modified Eagle's medium supplemented with 10% fetal bovine serum containing l-glutamine. Wild-type and mutant MC4R were transfected into HEK 293 cells with Lipofectamine (Invitrogen). For the internalization assays, stable cell lines expressing wild-type or RASSL-MC4R were generated by G418-mediated selection (10 µg/ml) of clones expressing the neomycin-resistance gene cotransfected with MC4R. Stable cell lines were maintained by selection with 5 µg/ml G418.

### cAMP Assays for MC4R Activity

cAMP production was measured with an ultra sensitive enzymatic cAMP assay (CatchPoint, Molecular Devices, Sunnyvale, CA). Transiently transfected cells were plated at 1×10^5^/well into 96-well plates coated with poly-D-lysine (Sigma). Forty-eight h after transfection, cells were rinsed in Krebs-Ringer bicarbonate buffer containing glucose (Sigma) and incubated in pre-stimulation buffer containing 0.75 mM 3-isobutyl-1-methylxanthine in the same buffer for 10 min at room temperature. Cells were then stimulated with forskolin, phosphate-buffered saline (basal conditions), various concentrations of α-, β-, and γ-MSH (natural agonists) or tetrahydroisoquinoline (THIQ, synthetic agonist) for 2 h at 37°C. The cells were lysed, and cAMP accumulation was measured according to the CatchPoint protocol. cAMP generated under the different experimental conditions was interpolated from a cAMP standard curve for each experiment. Four replicates were used for each condition, and all experiments were repeated at least three times. *E*max and EC50 values were calculated with SoftMax Pro. cAMP measurements were performed on transiently transfected cells.

### Detection of Membrane Expression of MC4R

Cell-surface expression of receptors was measured with an enzyme-linked immunosorbent assay (ELISA) that detects the extracellular FLAG tag. Transiently transfected cells were plated at 1×10^5^/well into 96-well plates coated with poly-D-lysine (Sigma). For internalization assays, cells were stimulated with the indicated concentrations of α-MSH or THIQ for the times indicated. Forty-eight h after transfection, cells were fixed in 4% paraformaldehyde (Sigma) for 10 min at 4°C. After two washes in phosphate-buffered saline, the cells were incubated in 1 µg/ml M1 anti-FLAG antibody (Sigma) for 2 h at room temperature, washed twice in phosphate-buffered saline, and incubated for 1 h at room temperature with horseradish peroxidase–conjugated goat-anti-mouse (1∶1000, BioRad, Chicago, IL). Cells were then washed three times in phosphate-buffered saline, and 0.2 ml of 2,2-azino-bis(3-ethylbenzthiazoline-6-sulfonic acid) liquid substrate (Sigma) was added to each well. After 15–60 min, the optical density was read at 405 nm in a spectrophotometer. Each experiment included four replicates per condition and was repeated at least three times.

## Results

### Strategy for MC4-RASSL Construction

**Figure 1:**
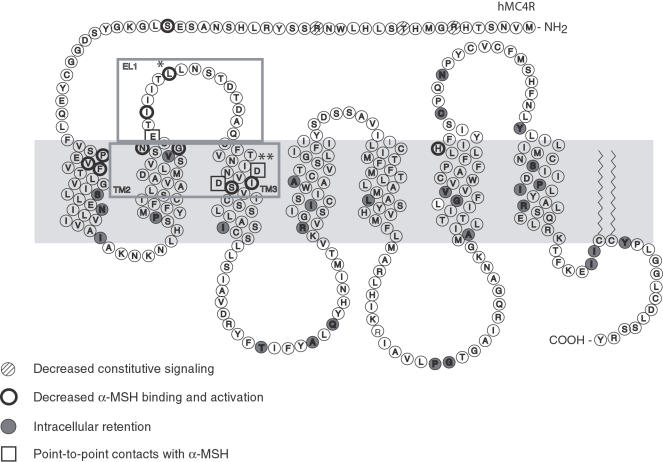
Model of the melanocortin-4 receptor. Boxed region indicates EL1, *L106P (Rm1), **D122A (Rm2).

Heterozygous point mutations in MC4R account for 1–6% of severe cases of human obesity. Over 50 different obesity-associated mutations have been described, most of which are missense mutations [23]–[27]. Point mutations in MC4R have been found in all the domains of the receptor (Fig. 1). Mutated receptors have impairments in ligand binding, internalization, or subcellular localization. All these mutations reduce the ability of the receptor to respond to α-MSH (data not shown and [26], [32]–[34]. In addition, structure-functions studies of MC4R have delineated the role of three key acidic residues (E100A, D122A and D126A) in transmembrane regions 2 and 3 (TM2 and 3) that are critical for α-MSH binding and activation [28], [29] (Fig. 1). By mapping the point mutations on MC4R and correlating them with specific defects in the receptor ([26], [32]–[34] and data not shown), we found that the majority of mutations buried in the TM regions and intracellular loops led to the intracellular retention of the receptor. To identify a receptor that was not activated by α-MSH, but still activated by a synthetic ligand, with minimal loss of membrane expression, we focused on five mutations (N97D, G98R, I102S, L106P and S127L) in extracellular loop 1 (EL1) and three in its flanking TM regions (E100A, D122A and D126A).

### Point Mutations in EL1 Do Not Alter the Cell-Surface Expression of MC4R

**Figure 2:**
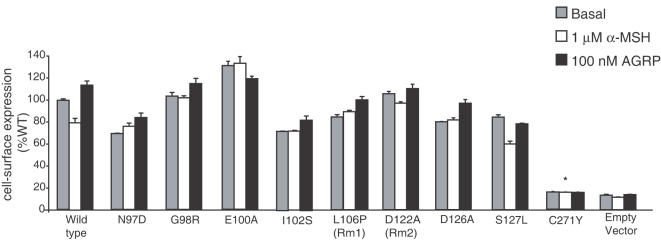
Cell-surface expression levels of wild-type and mutant MC4R were measured in transiently transfected HEK293 cells by ELISA. The prolactin signal sequence was added to the N-terminal domain of these receptors to ensure maximal membrane expression. MC4R mutation C271Y from TM6/EL3 significantly reduced membrane expression (*p*<0.05) and was used as a control. Empty vector (pcDNA3.1) was transfected as a negative control to detect background.

To examine the cell-surface expression of wild-type and mutant MC4R, we performed ELISA on transiently transfected HEK293 cells under basal conditions and after stimulation with α-MSH or AGRP. Wild-type MC4R was expressed on the cell surface and internalized after treatment with 1 µM α-MSH (Fig. 2), consistent with previous reports [35]. The inverse agonist AGRP stabilized receptor expression on the cell surface (Fig. 2). All point mutants examined, that is, N97D, G98R, E100A, I102S, L106P, D122A, D126A and S127L were relatively well-expressed at the cell surface (i.e., 70–120% of that seen with the wild-type receptor) (Fig. 2). With the exception of the S127L mutant, α-MSH was unable to internalize the mutant receptors, suggesting that the ligand fails to fully activate receptor signaling and initiate desensitization and internalization.

### MC4R EL1 Mutants Do Not Respond to α-MSH, but Do Respond to the Synthetic Ligand THIQ

**Figure 3:**
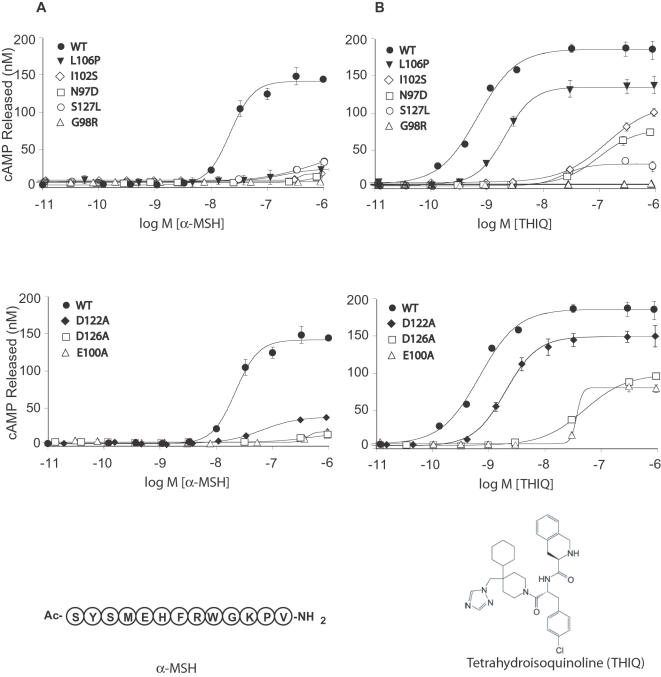
Wild-type (WT) and mutant MC4Rs were transiently transfected into HEK293 cells, which were stimulated with increasing concentrations of α-MSH *(A)* or of THIQ *(B)*, and cAMP was measured to generate dose-response curves. Data shown are mean±s.e.m. of quadruplicate determinations. The dose-response curves are representative of at least three independent experiments. The amino acid sequence of α-MSH and the chemical structure THIQ are shown below panels A and B, respectively.

HEK293 cells transiently transfected with wild-type or mutant MC4R were stimulated with increasing concentrations of α-MSH or THIQ. Wild-type MC4R was activated by α-MSH with an EC50 of 30.3 nM. Dose-response curves measuring cAMP production showed that all five obesity-associated human mutations in EL1, and point-to-point contact mutations in TM2 and TM3 significantly reduced α-MSH-mediated activation (Fig. 3A). The Emax of all mutated receptors was <5% to 40% of wild-type (Table 1); the EC50s of N97D, G98R, E100A, I102S, and L106P could not be determined. The EC50s for D122A, D126A, and S127L were in the mM range, well above the physiological range of α-MSH in vivo (Table 1). The loss of α-MSH-mediated activation in the human MC4R mutations is thought to be the basis of the obesity in patients carrying these mutations.

**Table 1:** Functional characterization of MC4R EL1 mutations.

Construct	Basal Activity (% of WT)	α-MSH activation		THIQ activation	
		*E*max (% of WT)	EC50 (nM)	*E*max (% of WT)	EC50 (nM)
WT MC4R	100	100	30.3±5.0	100	0.8±0.3
N97D	92.9±4.0	7.7±2.3	a	43.1±3.1	
G98R	99.3±19.2	2.4±0.8	a	2.6±0.6	a
E100A	123.0±3.4	13.6±2.2	a	44.54±4.9	a
I102S	89.8±1.1	11.9±1.6	a	49.2±2.2	a
L106P (Rm1)	71.2±6.8	10.2±0.5	a	73.4±14.8	2.4±0.9
D122A (Rm2)	185.8±22.8	19.8±1.2	a	90.3±6.2	2.9±0.5
D126A	69.6±7.5	21.3±0.4	a	50.7±3.6	a
S127L	149.5±13.3	38.3±2.0	a	21.7±3.5	a

Values are mean±s.e.m. (*n*≥3).

a EC50 could not be determined. WT, wild-type.

We next determined the ability of these mutated receptors to respond to the synthetic ligand THIQ, a selective MC4R agonist that potently stimulates cAMP production in the wild-type receptor in vitro [21] (EC50 = 3.5 nM) and the inhibition of food intake in vivo. THIQ was synthesized as a peptidomimetic of the potent α-MSH analog MT-II as a potential treatment for obesity [36] and is approximately 30-fold more potent at MC4R than α-MSH (Fig. 3 and Table 1). Interestingly, six of the seven mutations tested were rescued by THIQ, albeit to different degrees (Fig. 3B and Table 1). Notably, the human MC4R mutation L106P generated a robust cAMP response to THIQ, with an EC50 of 24.2 nM, similar to that of α-MSH at wild-type MC4R (EC50 = 30.3 nM). The D122A mutant, identified from structure-function studies of MC4R to be important for α-MSH binding [28], also responded robustly to THIQ (EC50 = 29.5 nM).

These experiments identified two RASSLs, L106P-MC4R and D122A-MC4R, which we designated RASSL MC4R 1 (Rm1) and RASSL MC4R 2 (Rm2), respectively. We further analyzed the potential for other amino acid substitutions (A, R, S, and W) at positions L106 and L107 to yield a more potent response to THIQ; however, these substitutions resulted either in mis-folded receptors that were not expressed at the cell surface or in receptors that did not show increased potency to THIQ (data not shown).

### Rm1 and Rm2 Have Different Basal Activities Compared to Wild-Type MC4R

**Figure 4:**
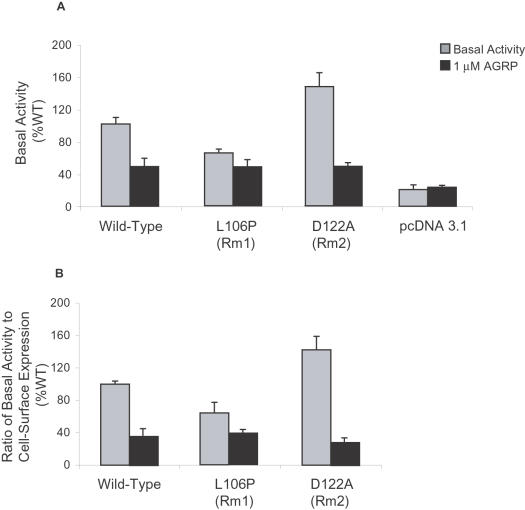
*A*, Basal activity of wild-type MC4R, Rm1 (L106P), Rm2 (D122A), and mock-transfected cells were measured by detecting basal cAMP release and cAMP release in response to AGRP. *B*, Ratios of cAMP accumulation to cell-surface expression for wild-type MC4R, Rm1, and Rm2 were determined in the same batch of transiently transfected cells. Values are means±s.e.m. of three independent experiments, each performed in quadruplicate.

MC4R has a well-documented intrinsic constitutive activity (measured by cAMP accumulation in cells transfected with MC4R in the absence of ligand) that can be suppressed by the inverse agonist AGRP [22] (Fig. 4 and Table 1). When we measured constitutive activity in all the EL1 mutants, we found that E100A, D122A, and S127L, had higher constitutive activity than the wild-type MC4R (Table 1). All mutants with increased basal activity were close to the EL1/TM2 or EL1/TM3 boundaries. All other mutants showed slightly lowered constitutive activity (Table 1) than wild-type MC4R. Rm1 had less AGRP-suppressible basal activity than wild-type MC4R (Fig. 4A), even though AGRP is a full antagonist when co-administered with THIQ, suggesting that Rm1 has lower intrinsic basal activity. Rm2 had a higher AGRP-suppressible basal activity than wild-type MC4R (Fig. 4A). AGRP alone had no effect on mock-transfected cells (Fig. 4A). The observed differences in basal activity might have reflected subtle differences in membrane expression. To exclude this possibility, we assessed basal activity by expressing constitutive activity (as judged by cAMP accumulation) as a ratio of cell-surface expression (as judged by ELISA) (Fig. 4B). Again, the variation in the basal activities of Rm1 and Rm2, compared to wild-type MC4R, were similar to those seen with direct cAMP measurements (Fig. 4A and B). Since constitutive signaling is important for normal GPCR function and altered basal activity can result in pathological disease states [37], [38], high-basal and low-basal RASSL variants could have specific applications for the study of the physiological role of constitutive signaling in vivo.

### Rm1 and Rm2 Do Not Respond to Other Related Endogenous Ligands

**Figure 5:**
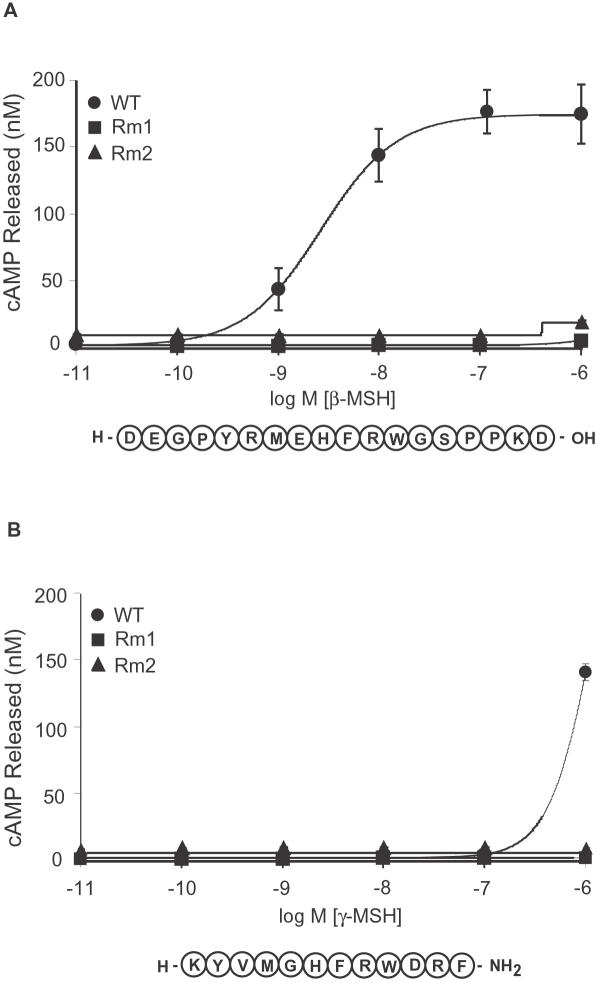
Wild-type MC4R, Rm1, and Rm2 were transiently transfected into HEK293 cells, which were stimulated with increasing concentrations of β-MSH *(A)* or of γ-MSH *(B)*, and cAMP was measured to generate dose-response curves. Data shown are mean±s.e.m. of quadruplicate determinations. The dose-response curves are representative of two independent experiments.

Proopiomelanocortin is a hypothalamic peptide that is post-translationally cleaved by proconvertases to release the melanocortin peptides α-, β- and γ-MSH, adrenocorticotropin and endorphins [39]. In addition to the well-established role of α-MSH in melanocortin receptor signaling, the related neuropeptides β-, and γ-MSH are also ligands in vitro for the melanocortin receptor family [40], [41], although their physiological roles in vivo are not well known. For full pharmacological control of G_s_ activation in vivo, it is important to ensure that β- and γ-MSH do not activate Rm1 and Rm2 in an uncontrolled manner. To this end, we measured cAMP release in transiently transfected cells expressing wild-type MC4R, Rm1, and Rm2 in response to increasing concentrations of β- and γ-MSH (Fig. 5). β-MSH elicited full activation of wild-type MC4R (EC50 = 2.78 nM), but no activation at Rm1 and Rm2 (Fig. 5A). γ-MSH did not activate Rm1 or Rm2 and elicited a response with significantly weaker potency from wild-type MC4R at high concentrations (1 µM; Fig. 5B).

### Internalization of MC4R, Rm1, and Rm2 in Response to α-MSH and THIQ

**Figure 6:**
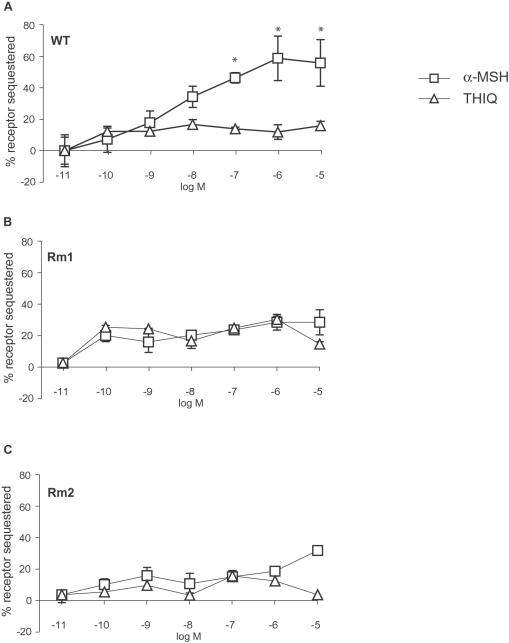
HEK293 cells stably expressing wild-type MC4R *(A)*, Rm1 (L106P) *(B),* and Rm2 (D122A) *(C)* were exposed to increasing concentrations of α-MSH or THIQ for 2 h. Cell-surface FLAG-tagged receptors were measured by ELISA. Receptor sequestration was calculated as the loss of receptor expression from the cell surface. Wild-type MC4R was internalized significantly more (*p*<0.05) in response to α-MSH than THIQ at doses of 10^−7^ M and higher. Data represent the mean±s.e.m. of two independent experiments, each performed in quadruplicate.

As another measure of receptor activation, we examined ligand-mediated internalization of MC4R, Rm1, and Rm2. We first generated HEK293 cell lines stably expressing FLAG-tagged wild-type MC4R and RASSLs. A 2-h exposure to increasing concentrations of α-MSH sequestered MC4R in a dose-dependent manner (maximal sequestration, ∼60%). MC4R was sequestered linearly over doses ranging from 1 nM to 1 µM (Fig. 6A), which correlates well with the range in which G_s_-mediated adenylyl cyclase activation is detected (Fig. 3A). Rm1 and Rm2 did not internalize in response to α-MSH, in keeping with the inability of α-MSH to activate these RASSLs (Fig. 6B and C). Surprisingly, a 2-h exposure to increasing concentrations of the synthetic ligand THIQ did not cause sequestration of either wild-type MC4R, Rm1, or Rm2 (Fig. 6A, B, C), even though similar concentrations of THIQ activated both Rm1 and Rm2 as measured by cAMP accumulation (Fig. 3).

**Figure 7:**
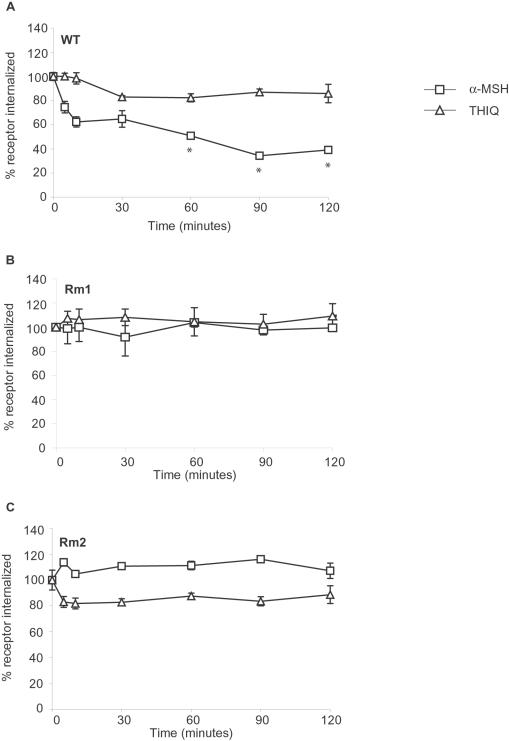
HEK293 cells stably expressing wild-type MC4R *(A)*, Rm1 *(B),* and Rm2 *(C)* were exposed to 1 µM α-MSH or THIQ for the times indicated. Cell-surface FLAG-tagged receptors were measured by ELISA. Receptor internalization was calculated as the loss of receptor expression from the cell surface. After 60 min, significantly more wild-type MC4R was internalized (*p*<0.05) in response to α-MSH than THIQ. Data represent the mean±s.e.m. of two independent experiments, each performed in quadruplicate.

We next examined the kinetics of internalization of MC4R as measured by the loss of cell-surface MC4R expression. Treatment of cells stably expressing MC4R with 1 µM α-MSH resulted in the time-dependent internalization of the wild-type receptor. About 40% of receptors were internalized within 10 min and about 20% over the next 80 min (Fig. 7A and [35]. After 4 h, the amount of MC4R on the cell surface did not decrease further (not shown), indicating that the rate of internalization of MC4R is at equilibrium with the rate of MC4R cell-surface expression. As in the dose-response experiments (Fig. 6), addition of 1 µM THIQ did not result in internalization of wild-type MC4R during a 2-h exposure (Fig. 7A). Rm1 and Rm2 were not internalized in response to α-MSH during either a 2-h (Fig. 7B and C) or 4-h exposure (not shown). Although Rm1 did not internalize in response to 1μM THIQ, we observed an approximately 20% decrease in the cell-surface expression of Rm2 in response to the same concentration of THIQ.

## Discussion

We developed two G_s_-coupled RASSLs based on the MC4R. Rm1 and Rm2 were generated by identifying mutations in MC4R that were unresponsive to the endogenous ligand α-MSH but were still fully responsive to the high-affinity MC4R-selective synthetic ligand THIQ. Rm1 and Rm2 are well-expressed at the cell-surface and do not respond to other endogenous ligands, such as β- and γ-MSH. This and other studies [28], [32] indicate that EL1, TM2, and TM3 are critical regions for α-MSH-mediated activation of MC4R; however, the small molecule THIQ was able to elicit at least a partial response when key residues in these regions are mutated. An important difference between the two RASSLs reported here lies in their intrinsic constitutive activities. Rm1 has a low constitutive activity (75% of wild-type), and Rm2 has high constitutive activity (140% of wild-type). This variation may be particularly advantageous for studying the physiological consequences of constitutive signaling. For example, loss of basal activity of MC4R is associated with human obesity (Srinivasan et al., 2004), and increased basal G_s_ signaling has been associated with increased cell proliferation and tumor formation in the pituitary [37] and other endocrine glands [42]. Thus, the ability to modulate basal signaling offers a unique opportunity to separate the physiological contribution of basal signaling versus ligand-mediated GPCR activation *in vivo*. In addition, since THIQ is able to elicit a partial response from some mutated MC4Rs, our findings suggest that THIQ may be a potential obesity therapeutic for people carrying MC4R mutations.

The differences in internalization of wild-type MC4R, Rm1, and Rm2 in response to α-MSH and THIQ are particularly interesting. Dose-response curves measuring adenylyl cyclase activation and cAMP generation showed that THIQ is 30-fold more potent than α-MSH at MC4R. This increased potency may reflect the lower internalization rate of MC4R in response to THIQ than to α-MSH. Further studies on agonist-selective internalization of MC4R will be required to elucidate the precise mechanistic explanation for these effects. Agonist-selective internalization has certainly been demonstrated for other GPCRs. For example, the G_i_-coupled κ-opioid receptor is internalized within 30 min in response to physiological concentrations of the alkaloid agonist etorphine and its synthetic analog, DAMGO [43]. However, it is insensitive to internalization by the analgesic morphine at concentrations far greater than required to inhibit adenylyl cyclase via the mu opioid receptor. It will be interesting to further explore the molecular basis of agonist-selective internalization at MC4R and to identify potential differences in phosphorylation by GRKs or arrestin binding that underlie this observation.

The concept of designing engineered receptors as tools to control signal transduction pathways *in vivo* was first used to make “designer dimerizers” [44]. An intracellular growth factor receptor domain was fused to an extracellular drug-binding protein domain. The binding of a synthetic chemical dimerizer resulted in the reversible dimerization of the fusion protein, which then triggers a cascade of downstream signaling events. This system has been successfully used to control proliferation in many cell types, including myoblasts, hepatocytes, and hematopoetic stem cells [44]–[46]. For signaling engineering in GPCRs, a designer β-adrenergic receptor was developed by Small and colleagues [47]. A synthetic ligand-binding site was engineered into TM3 of the receptor, and the C-terminal tail was truncated and fused to the G_αs_ protein. The two-way selection this provided allowed targeted activation of the receptor upon ligand binding. Although elegant, the system was of limited use because the potency of the ligand was too low (EC50 in µM range) for systemic administration of the ligand in mice. The RASSL concept circumvents the task of engineering a new ligand binding site, since preexisting high-affinity ligands are used to activate these receptors *in vivo*. In addition to the G_i_ and G_s_ RASSLs developed in our laboratory [15], a G_s_-coupled RASSL was recently developed using the 5HT-4 receptor [14]. It will be interesting to compare G_s_-mediated signaling from the RASSLs based on the 5HT-4 receptor and the MC4R for receptor-specific differences in G_s_ protein activation in the same cell types *in vivo*.

The most immediate application of the MC4R-based RASSLs lies in their use as G_s_ switches for studies of food-intake and body-weight regulation in mice. Experiments based on peripherally administered α-MSH have delineated a role for MC4R in these processes [19]. However, a detailed, mechanistic understanding of the cellular and biochemical changes in the hypothalamus in response to MC4R signaling has been difficult to elucidate because of the presence of the endogenous hormone, α-MSH, and MC3R (also activated by α-MSH), which may contribute to the maintenance of energy homeostasis [48]–[50]. The observation that loss-of-function mutations in MC4R are often correlated with early-onset obesity [32] suggests that MC4R plays a role during neonatal life, however its selective role in adulthood can be evaluated in MC4-RASSL knock-in transgenic mice. In addition, the recent demonstration of the trophic actions of leptin in mediating synapse formation and plasticity in the hypothalamus [51] suggests that MC4R, a critical downstream component of the leptin pathway, may mediate these effects. A targeted gene knock-in approach in mice could be used to replace the genomic copies of the endogenous receptor with Rm1 or Rm2. Alternatively, retrovirus-mediated hypothalamic expression of RM1 and Rm2 in adult MC4R-deficient mice could be used as a first step in evaluating Rm1 and Rm2 in vivo. Since Rm1 possesses low basal activity, it would produce less cAMP than the wild-type receptor, and the phenotype of the mice expressing Rm1 (predicted to be overweight compared to wild-type mice) can be used to determine the functional contribution of MC4R basal signaling. Conversely, Rm2 has high basal signaling and would be predicted to reduce body weight. Administration of THIQ can then be used to separate the effects of ligand-mediated versus basal signaling in the specific MC4R-expressing neurons at different times during pre- and postnatal development to elucidate the contribution of MC4R-mediated G_s_ signaling in synaptic plasticity and other downstream events required to maintain energy homeostasis. Having two RASSLs with different basal activities would be especially useful because in vitro and indirect in vivo [52] evidence suggest that the basal activity of MC4R may also be important these processes.

Traditional biochemical studies of receptor function have used site-directed or saturation mutagenesis to assess structure-function relationships for the role of individual amino acids in conserved protein domains, and such studies have been very useful towards our current understanding of protein function. Novel strategies using *in silico* methods that employ the knowledge of coevolved energetically coupled residues have also yielded valuable information on functionally important surfaces required for ligand binding in receptors of previously unknown function [53], [54]. The study presented here has hinged on the availability of human genetic data, where substitution of functionally important residues is correlated with a loss-of-function disease phenotype. In the absence of high-resolution structural information for GPCRs, this knowledge rapidly allows one to target those regions most likely to be useful for receptor engineering. Even though the MCR4 mutations we studied are relatively rare (∼5% of severe obesity), the obesity phenotype allowed them to be identified and rapidly tested for the biochemical consequences of the mutation. As we gain a better understanding of genotype-phenotype correlations for other genes, it may be possible to find other collections of rare point mutations that result in human phenotypic variation. The MC4R provides a model of how a detailed understanding of human genetics can be used as a powerful tool for protein engineering.
